# Machine Learning Models for Frailty Classification of Older Adults in Northern Thailand: Model Development and Validation Study

**DOI:** 10.2196/62942

**Published:** 2025-04-02

**Authors:** Natthanaphop Isaradech, Wachiranun Sirikul, Nida Buawangpong, Penprapa Siviroj, Amornphat Kitro

**Affiliations:** 1 Department of Community Medicine, Faculty of Medicine, Chiang Mai University, 110, Intrawarorot Road, Meaung, 50200, Thailand, 66 53935472, 66 935476; 2 Environmental and Occupational Medicine Excellence Center, Faculty of Medicine, Chiang Mai University, Chiang Mai, Thailand; 3 Center of Data Analytics and Knowledge Synthesis for Health Care, Faculty of Medicine, Chiang Mai University, Chiang Mai, Thailand; 4 Department of Biomedical Informatics and Clinical Epidemiology, Faculty of Medicine, Chiang Mai University, Chiang Mai, Thailand; 5 Department of Family Medicine, Faculty of Medicine, Chiang Mai University, Chiang Mai, Thailand

**Keywords:** aged care, gerontology, geriatric, old, aging, clinical decision support, delivering health information and knowledge to the public, diagnostic systems, digital health, epidemiology, surveillance, diagnosis, frailty, machine learning, prediction, predictive, AI, artificial intelligence, Thailand, community dwelling, health care intervention, patient care

## Abstract

**Background:**

Frailty is defined as a clinical state of increased vulnerability due to the age-associated decline of an individual’s physical function resulting in increased morbidity and mortality when exposed to acute stressors. Early identification and management can reverse individuals with frailty to being robust once more. However, we found no integration of machine learning (ML) tools and frailty screening and surveillance studies in Thailand despite the abundance of evidence of frailty assessment using ML globally and in Asia.

**Objective:**

We propose an approach for early diagnosis of frailty in community-dwelling older individuals in Thailand using an ML model generated from individual characteristics and anthropometric data.

**Methods:**

Datasets including 2692 community-dwelling Thai older adults in Lampang from 2016 and 2017 were used for model development and internal validation. The derived models were externally validated with a dataset of community-dwelling older adults in Chiang Mai from 2021. The ML algorithms implemented in this study include the k-nearest neighbors algorithm, random forest ML algorithms, multilayer perceptron artificial neural network, logistic regression models, gradient boosting classifier, and linear support vector machine classifier.

**Results:**

Logistic regression showed the best overall discrimination performance with a mean area under the receiver operating characteristic curve of 0.81 (95% CI 0.75‐0.86) in the internal validation dataset and 0.75 (95% CI 0.71‐0.78) in the external validation dataset. The model was also well-calibrated to the expected probability of the external validation dataset.

**Conclusions:**

Our findings showed that our models have the potential to be utilized as a screening tool using simple, accessible demographic and explainable clinical variables in Thai community-dwelling older persons to identify individuals with frailty who require early intervention to become physically robust.

## Introduction

The world population is moving toward an aging society. As health care technology improves, people are expected to live longer and healthier [1]. According to the World Health Organization, the population aged ≥60 years will increase from 1 billion in 2020 to 2.1 billion in 2050 and the number of people aged ≥80 years will reach 426 million in 2050 [2]. Researchers predicted that the proportion of people in Thailand aged ≥60 years would be more than 20% of the population in 2025 and more than 30% in 2031 [3,4].

The prevalence of frailty is high among older adults aged ≥60 years [5]. Global frailty prevalence ranges from approximately 10% to 12% [6-11]. The percentage varies by age, gender, and frailty classification tool. In Thailand, frailty prevalence was 22.1%, which is twice the global frailty prevalence, according to the Thai National Health Examination Survey cohort in 2018. Specifically, Thailand’s northern region frailty prevalence was found to be 15% [12,13]. This creates concerns about the increasing aging population in Thailand.

Frailty is defined as a clinical state of increased vulnerability due to the age-associated decline of an individual’s body resulting in increased morbidity and mortality when exposed to everyday or acute stressors [14,15]. This clinical syndrome is associated with decreased quality of life [7], slow gait speed [16], more depressive symptoms, higher BMI, reduced cognitive function [17], decreased strength [12], and increased risk of fall, hospital re-admission, and all-cause mortality in the older adult population [13,18-20]. Frailty has become a crucial research topic because this clinical syndrome can be reversed. Studies have shown that early detection and intervention can revert individuals from a frail to a fit state [21-23].

However, incorporating frailty evaluation into clinical practice in a primary care context is challenging due to increased administrative tasks, time limitations, and a lack of diagnostic effectiveness [15,24,25]. In the age of technology, health informatics has become an important role in health care research [26,27]. Several studies have applied information technology to frailty detection in primary care settings using machine learning models and artificial intelligence [28-30]. A study from Canada showcased an efficient frailty identification tool using the XGBoost machine learning model. Features used for the model were medication, medical billing codes, and other primary care clinical data [31]. Another example showed the development of a predictive machine learning model for frailty conditions based on a database of demographic data and clinical characteristics [32]. In China, a study simplified the Frailty Index assessment for older individuals using machine learning techniques and showed that logistic regression was the best performing and most interpretable model, with a mean area under the receiver operating characteristic curve (AUC) of 0.974 in the internal validation dataset [33]. Another study in China also developed and validated models using data from 6997 older adult participants to predict frailty risk, with random forest (RF) and logistic regression (LR) achieving AUC values of 0.77 and 0.76, respectively [34].

Despite the abundance of evidence of conducting frailty assessments using machine learning globally and in Asia, we have found no integration of data science tools and frailty screening and surveillance studies in Thailand. Most studies focused on the risk factors and their association with frailty syndrome but failed to show application in real-world settings [12,13,35,36]. This leads to our research question, “Can machine learning models predict frailty in community-dwelling older adults in Northern Thailand?” Therefore, we propose an approach to frailty detection using a machine learning model in the community-dwelling population from Lampang and Chiang Mai, Thailand, to effectively screen frailty status among Thai community-dwelling older adults and to help decrease clinicians’ burden of work.

## Methods

### Source of Data

#### Development and Internal Validation Datasets

The datasets were derived from a cross-sectional study carried out in Lampang in 2016 and 2017; it is a northern Thai province with one of the highest aging indexes [37]. This study included older adults aged ≥60 years. Those with dementia (as determined by the Thai Mental State Examination), blindness, deafness, bedridden status, disabilities, or severe acute diseases were excluded.

To represent urban (8 villages), semiurban (8 villages), and rural (8 villages) communities, 24 villages in 3 districts were chosen. The records of the primary care unit were used to compile lists of community-dwelling older adults. The details of the data collection in this study are published elsewhere [36]. A total of 2228 older adults from this study were derived for model development and internal validation.

#### External Validation Dataset

The datasets used for external validation were derived from a cross-sectional study in Kuamung (suburban), Sankampang, Chiang Mai, Thailand, in 2021. The participants were included and excluded from the study with the same criteria as described in the internal validation datasets. A total of 464 older adults from this study were used for external validation of the derived models.

### Predictors

#### Characteristics and Demographics

Assessments were conducted through a questionnaire at the participant’s residence. The questionnaire included questions about sociodemographic information (age, household living arrangement, gender, and education level), self-reported medical diagnoses (such as hypertension, diabetes mellitus, and heart disease), level of physical activity per week, and exhaustion.

#### Anthropometric Variables

Anthropometric measures included BMI, waist circumference, and calf circumference (CC). A handheld dynamometer (Takei TKK5001) was used to assess handgrip strength. Height and calf circumference were measured with standard tape (Tajima brand, PIT-20BL model), and weight was measured with a calibrated weighing scale (Shaper Disney). The CC was measured over the unclothed area at the maximum diameter on the left leg. The tape was wrapped snugly around the calf and measured to the nearest 0.1 cm. All measurements were administered by 10 qualified field investigators, and the measurements were standardized by the principal investigator.

#### Outcome Variable

Frailty was evaluated based on Fried’s phenotype [38], which includes the following five criteria:

1. Unintentional weight loss: The participant will be asked “In the last ten years, have you lost more than 10 pounds intentionally (not due to diet or exercise)?” If yes, then frail for weight loss criterion. At follow-up, weight loss was calculated as: (weight in previous year – current measured weight)/(weight in previous year)=K. If K≥0.05 and the subject did not report that he/she was trying to lose weight (ie, unintentional weight loss of at least 5% of previous year’s body weight), then frail for weight loss=yes.
2. Exhaustion, assessed with self-report using Fried’s method of assessment. The participant was first asked to self-assess whether she/he felt exhausted. If yes, she/he would be asked to rate the severity of the exhaustion. Ratings of 2-4 suggested a positive assessment.
3. Physical activity, based on the short version of the Minnesota Leisure time Activity questionnaire, which asked about walking, chores (moderately strenuous), mowing the lawn, raking, gardening, hiking, jogging, biking, exercise cycling, golf, single tennis, doubles tennis, racquetball, and calisthenics. For men, those with <383 kcals of physical activity per week were frail. For women, those with <270 kcals of physical activity per week were frail.
4. Walk time, stratified by gender and height (gender-specific cutoff for medium height).
  - Height ≤173 cm and walk time ≥7 seconds for men.
  - Height >173 cm and walk time ≥6 seconds for men.
  - Height ≤159 cm and walk time ≥7 seconds for women.
  - Height >159 cm and walk time ≥6 seconds for women.
5. Grip strength, stratified by gender and BMI quartiles.
  - For men:
    - BMI ≤24 and grip strength ≤29 for men
    - BMI 24.1‐26 and grip strength ≤30
    - BMI 26.1‐28 and grip strength ≤30
    - BMI >28 and grip strength ≤32
  - For women:
    - BMI ≤23 and grip strength ≤17
    - BMI 23.1-26 and grip strength ≤17.3
    - BMI 26.1-29 and grip strength ≤18
    - BMI <29 and grip strength ≤21

Older adults with 3 or more phenotypes were considered to have physical frailty, and those who had 1 or 2 phenotypes were classified as prefrailty. According to the study design of the derived datasets, the outcome was concurrently measured with the predictors.

#### Sample Size Calculation

The sample size for model development was calculated using the *pmsampsize* package via STATA (version 16; StataCorp). A minimum sample size required 16.41 events per predictor, which was 1519 patients with 263 frailty cases based on a maximum candidate predictor of 16, frailty prevalence of 17.29% in the dataset for model development, small overfitting defined as expected shrinkage of predictor effects by 10% or less, a small difference in the developed model’s apparent and optimism-adjusted values of 0.15 (*R*^2^_Nagelkerke_), as suggested by Riley et al [39]. For the external validation, the sample size was calculated using the *pmvalsampsize* package via STATA. From the available data of 464 patients with 192 frailty cases, it was able to precisely estimate confidence interval widths of observed/expected statistic of 0.22, calibration slope of 0.60, and concordance statistic of 0.10 [40].

### Statistical Analysis Methods and Synthesis of the Results

Data exploration was performed using descriptive statistics to determine the data quantity, quality, and distribution. A frequency and a percentage were used to describe categorical variables. For continuous variables, a mean with SD was used for parametrically distributed data, and a median with an IQR was used for nonparametrically distributed data. The comparison of characteristics between the frail and nonfrail populations was performed using the chi-square test for categorical variables, the independent *t* test for parametric variables, and the rank-sum test for nonparametric variables. The methods for feature selection, model development, and validation are described in the section “Model Development and Validation”. Estimates of model discrimination and optimism are reported as the mean AUC with 95% CI across all repetitions of cross-validation for the internal validation and the AUC with 95% CI from 1000 bootstrapping samples for the external validation. To further explain model performance, we also created model calibration plots and calculated secondary metrics of prediction models, including the confusion matrix and specificity, sensitivity, and predictive values.

### Missing Data and Imputation

There was only missing data in participants' age in the internal validation dataset (4/2228, 0.18%); therefore, a complete case analysis was performed on the dataset. The external validation dataset contained missing data, including age (2/464, 0.43%), BMI (11/464, 2.37%), waist and calf circumference (3/464, 0.64%), handgrip strength (2/464, 0.43%), and frailty status (4/464, 0.86%). We performed multiple imputations of missing data, except for frailty status (the outcome of interest), using the predictive mean matching imputation with 5 nearest neighbors via the *KNNImputer* from the *Scikit-Learn library 1.1.2.* Four participant records that did not have frailty status were removed (list-wise deletion).

### Model Development and Validation

#### Feature Selection

The classification models were developed using the variables from the derived datasets. The variables for model development were selected using both a data-driven method and domain expertise. For a data-driven selection, a multivariable logistic regression with stepwise backward elimination was performed to determine statistically significant variables (*P* value less than .20). For the *P* value threshold of .20, we used a higher threshold to give priority to clinical reasoning in selecting variables by domain expertise and associated factors from the previous studies, along with statistical significance, which would allow more important variables to be entered into the model.

#### Rebalancing Data Strategy

We resolved unbalanced classification for the dataset’s minority class by using the Synthetic Minority Oversampling Technique (SMOTE) to enhance model decision boundaries via the *imblearn.over_sampling.SMOTE* package [41,42].

### Model Development and Internal Validation

The model development and validation were performed using Python (version 3.9; Python Software Foundation). The machine learning algorithms implemented in this study include the k-nearest neighbors (KNN) algorithm, RF machine learning algorithms, multilayer perceptron artificial neural network (MLP), gradient boosting classifier (GBC), linear support vector machine classifier (SVM), and LR models via the *Scikit-Learn library 1.1.2*. The hyperparameters were determined by using a grid search via the *GridSearch CV* package with 10-fold cross-validation on the derived dataset to determine the parameters of each model that led to the best discriminative performance. For 10-fold cross-validation, the derived dataset was divided into 10 folds of data and repeated 10 times to perform model training and testing. For each iteration, 9 folds of data were used to train the model and then it was tested with the remaining fold to ensure that almost all the derived data were used to train and test the models. The discriminative performance of the derived models was assessed by computing a confusion matrix and sensitivity, specificity, and predictive values, as well as AUC with a 95% CI.

The model calibration was evaluated using the calibration plot, which indicated the congruence between the observed proportion of the actual probability of outcome and the mean predicted probability (MPP) from the derived models.

### External Validation

The derived models were validated again with the external validation dataset to determine the model optimism and calibration. The discriminative performance and the model optimism were re-evaluated and presented by discriminative performance matrices and a 95% CI of AUC from the 1000 bootstrapping samples, respectively. The model calibration using the external validation dataset was re-evaluated using the calibration plot.

### Ethical Considerations

This study complies with the research with exemption category and has been certified by the Research Ethics Committee of the Faculty of Medicine, Chiang Mai University (study code: COM-2565‐09159, number EXEMPTION 9159/2022). We requested a waiver of informed consent because this study is a retrospective analysis that exclusively utilizes anonymized secondary data from our research database, without collecting any additional information from medical records or other sources. All participants have previously provided informed consent for the primary data collection as described elsewhere [35,36,43]. All personal patient data were anonymized by removing citizen ID numbers, hospital numbers, addresses, and contact information from the dataset. The investigator cannot trace or identify individuals. The study results were reported in accordance with the Transparent Reporting of a multivariable prediction model for Individual Prognosis Or Diagnosis plus Artificial Intelligence (TRIPOD+AI) statement [44].

## Results

### Baseline Characteristics of Participants in Each Dataset

Baseline characteristics of participants in this study are shown in Table 1. The participants in the internal validation dataset had a mean age of 71.0 years. Most of the participants were male and had finished primary school. Among 2228 old adults, 2160 lived with either their spouse, relative, or children while the others were living alone. The average BMI of the participants was 32.6 (SD 7.4) kg/m^2^. The prevalence of hypertension, dyslipidemia, type 2 diabetes mellitus, and heart disease were 45.26%, 19.67%, 16.21%, and 4.31%, respectively. The averages of waist circumference, calf circumference, handgrip strength, and walk time were 83.40 (SD 11.23) cm, 32.40 (SD 4.35) cm, 32.40 (SD 6.68) kg, and 6.42 (SD 2.01) minutes, respectively. Overall, 9.79% of the participants were exhausted and 16.3% had physical activity higher than 150 minutes per week. The prevalence of frailty was 17.3% (n=385).

**Table 1.** The characteristics of participants in the development and internal validation datasets and the external validation dataset.

Characteristics	Development and internal validation datasets (Lampang, 2016‐2017; N=2228)	External validation datasets (Chiang Mai, 2021; N=464)	*P* value
Age (years), mean (SD)	70.96 (7.49)	70.68 (5.58)	.45
**Gender, n (%)**			
Male	1569 (70.45)	193 (41.59)	<.001
Female	658 (29.55)	271 (58.41)	
**Household living arrangement, n (%)**			
Living alone	160 (7.18)	42 (9.05)	<.001
Living with spouse	1177 (52.85)	9 (1.94)	
Living with children	823 (36.96)	283 (60.99)	
Living with relatives or others	67 (3.01)	130 (28.02)	
BMI (kg/m^2^), mean (SD)	32.64 (7.40)	22.73 (3.89)	.001
**Education, n (%)**			
No education	192 (8.62)	12 (2.61)	<.001
Primary school	1745 (78.36)	398 (86.52)	
Secondary school or higher	290 (13.02)	50 (10.87)	
**Underlying diseases, n (%)**			
Hypertension	1008 (45.26)	237 (51.08)	.02
Dyslipidemia	438 (19.67)	79 (17.03)	.19
Type 2 diabetes mellitus	361 (16.21)	78 (16.81)	.75
Heart diseases	96 (4.31)	18 (3.88)	.68
**Anthropometric variables, mean (SD)**			
Waist circumference (cm)	83.40 (11.23)	81.16 (10.80)	<.001
Calf circumference (cm)	32.40 (4.35)	32.85 (4.55)	.04
Walk time (min), mean (SD)	6.42 (2.01)	8.50 (5.38)	<.001
Exhaustion, n (%)	218 (9.79)	189 (40.74)	<.001
Adequate level of physical activity as defined by the World Health Organization, n (%)	363 (16.30)	104 (22.42)	<.001
Grip strength (kg), mean (SD)	32.40 (6.68)	19.87 (7.29)	<.001
Frailty, n (%)	385 (17.29)	192 (41.74)	<.001

In the external validation dataset, the participants had a mean age of 70.68 years. Most of the participants were male and had finished primary school. Among 464 persons, 422 lived with either their spouse, relative, or children, while the others were living alone. The average BMI of the participants was 22.73 (SD 3.89) kg/m^2^. The prevalence of hypertension, dyslipidemia, type 2 diabetes mellitus, and heart disease were 51.08%, 17.03%, 16.81%, and 3.88%, respectively. The averages of waist circumference, calf circumference, grip strength, and walk time were 81.16 (SD 10.80) cm, 32.85 (SD 4.55) cm, 19.87 (SD 7.29) kg, and 8.50 (SD 5.38) minutes, respectively. The prevalence of participants with exhausted state was 40.74%, and 22.42% of the participants participated in physical activity more than 150 minutes per week.

Comparing the variables between the 2 datasets, we found that gender, household living arrangement, BMI, education, waist circumference, walk time, exhaustion, grip strength, and level of physical activity were significantly different.

### Model Development

We used a dataset from Lampang (2016‐2017; N=2228) for model development. The association between candidate predictors and frailty by univariate analysis is reported in Table 2. Feature selection for model development was selected by a backward elimination approach via a multivariable logistic regression and expert judgment. We chose the following features as model predictors: age, gender, status, underlying diseases (hypertension and dyslipidemia), BMI, waist and calf circumference, and level of exhaustion. Finally, the derived data included 385 participants with frailty and 1842 participants without frailty, and all candidate predictors, as shown in Table 2, were used in model development.

**Table 2.** The comparison of participants’ characteristics and their associations with frailty in the development and internal validation datasets.

Characteristics	Adjusted odds ratio – full model (95% CI)	*P* value	Adjusted odds ratio – reduced model (95% CI)	*P* value
Age (years)	1.04 (1.02‐1.07)	<.001	1.09 (1.06‐1.11)	<.001
**Gender**				
Male	Reference		Reference	
Female	1.93 (1.26‐2.94)	.002	0.90 (0.63-1.27)	.55
**Status**				
Living alone	Reference			
Living with others	0.92 (0.46‐1.86)	.82	0.87 (0.45‐1.68)	.68
BMI (kg/m^2^)	0.92 (0.90‐0.95)	<.001	0.91 (0.88‐0.93)	<.001
**Education**				
No education	Reference			
Primary school	1.00 (0.59‐1.70)	.99		
Secondary school or higher	1.34 (0.65‐2.77)	.42		
**Underlying diseases**				
Hypertension	1.47 (1.03‐2.10)	.03	1.27 (0.92‐1.75)	.15
Dyslipidemia	1.22 (0.78‐1.91)	.38	1.45 (0.97‐2.17)	.07
Type 2 diabetes mellitus	0.84 (0.54‐1.31)	.44		
Heart disease	1.03 (0.45‐2.34)	.95		
**Anthropometric variables**				
Waist circumference (cm)	1.02 (1.00‐1.04)	.046	1.02 (1.00‐1.04)	.02
Calf circumference (cm)	1.05 (1.01‐1.09)	.01	1.04 (1.01‐1.08)	.02
Walk time (min)	1.89 (1.71‐2.10)	<.001	1.95 (1.78‐2.13)	<.001
Exhaustion	20.39 (12.61‐32.94)	<.001	28.23 (18.22‐43.73)	<.001
Adequate level of physical activity as defined by the World Health Organization	4.49 (3.06‐6.59)	<.001		
Grip strength	0.83 (0.79‐0.85)	<.001		

However, the model appeared to be poor in frailty prediction performance and imbalanced as the classifiers intended to classify only the majority class (accuracy paradox). Therefore, a rebalancing strategy by SMOTE was applied to counter this problem. The oversampling data were generated and rebalanced the minority group in a 1:1 ratio. The details of the final model’s hyperparameters using *GridSearch CV* are presented in Table S1 in Multimedia Appendix 1.

Adjusted odds ratios (aORs) of the full model were obtained from a multivariable logistic regression with all features. aORs of the reduced model were obtained from a multivariable logistic regression with stepwise backward elimination (*P*<.10) and feature selection based on the domain expertise.

### Discrimination Performance of Internal Validated Models

**Figure 1.**
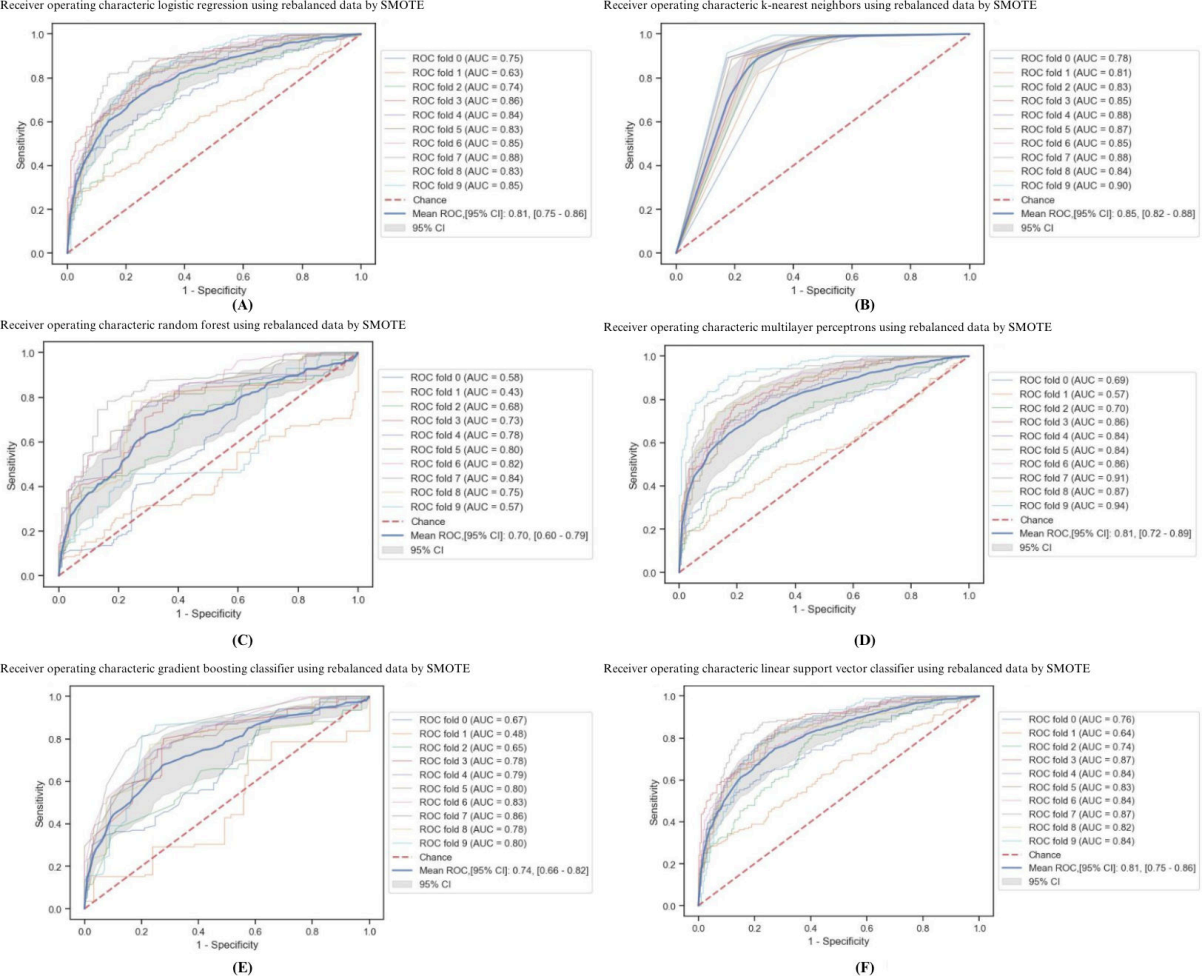
Receiver operating characteristic curves from 10-fold cross-validation of the rebalanced learning classifiers by SMOTE: (A) logistic regression model: mean AUC 0.81 (95% CI 0.75‐0.86); (B) k-nearest neighbors model: mean AUC 0.85 (95% CI 0.82‐0.88); (C) random forest model: mean AUC 0.70 (95% CI 0.60‐0.79); (D) multilayer perceptron model: mean AUC 0.81 (95% CI 0.72‐0.89); (E) gradient boosting classifier model: mean AUC 0.74 (95% CI 0.66‐0.82); (F) linear support vector machine classifier model: mean AUC 0.75 (95% CI 0.75‐0.86). ROC: receiver operating characteristic curve; SMOTE: synthetic minority oversampling technique.

We evaluated the model’s performance by 10-fold cross-validation. The discrimination performances of the models are presented in Table 3 and Figure 1. The overall discrimination performance was presented by mean AUC 10-fold cross-validation. The KNN model achieved the highest overall performance with a mean AUC of 0.85 (95% CI 0.82‐0.88), followed by MLP (AUC 0.81, 95% CI 0.72‐0.89), LR (AUC 0.81, 95% CI 0.75‐0.86), and SVM (AUC 0.75, 95% CI 0.75‐0.86), respectively. In addition, the KNN model had the highest sensitivity and specificity (89% and 76%, respectively). RF had the lowest discrimination performance with almost all metrics. Other metrics that were not affected by data rebalancing, like positive predictive value (PPV) and negative predictive value (NPV), were also used to express the performance of the models. The best performances for both PPV and NPV were found in the KNN model (0.79 and 0.87, respectively).

**Table 3.** Discrimination and optimism of internal validated models with 95% CIs.

Models	AUC^a^, mean (95% CI)	Predictive values		Sensitivity	Specificity
		Positive	Negative		
LR^b^	0.81 (0.75‐0.86)	0.74 (0.68‐0.80)	0.74 (0.69‐0.78)	0.74 (0.69‐0.79)	0.73 (0.65‐0.81)
KNN^c^	0.85 (0.82‐0.88)	0.79 (0.76‐0.82)	0.88 (0.86‐0.90)	0.89 (0.87‐0.91)	0.76 (0.71‐0.81)
RF^d^	0.70 (0.60‐0.79)	0.65 (0.58‐0.73)	0.70 (0.63‐0.76)	0.79 (0.74‐0.83)	0.55 (0.39‐0.71)
MLP^e^	0.81 (0.72‐0.89)	0.73 (0.64‐0.81)	0.75 (0.68‐0.81)	0.78 (0.71‐0.84)	0.69 (0.57‐0.81)
GBC^f^	0.74 (0.72‐0.89)	0.66 (0.59‐0.74)	0.70 (0.63‐0.78)	0.78 (0.73‐0.83)	0.58 (0.43‐0.73)
SVM^g^	0.75 (0.75‐0.86)	0.74 (0.68‐0.80)	0.73 (0.69‐0.78)	0.74 (0.69‐0.79)	0.73 (0.65‐0.81)

a AUC: area under the receiver operating characteristic curve.

b LR: logistic regression.

c KNN: k-nearest neighbors.

d RF: random forest.

e MLP: multilayer perceptron artificial neural network.

f GBC: gradient boosting classifier.

g SVM: linear support vector machine classifier.

### Discrimination Performance of External Validated Models

We validated our trained model with an external validation dataset to evaluate the bias and variance of our trained models. SMOTE was not applied to the dataset since it was already well-balanced between participants with and without frailty. The performance of machine learning models validated by the external validation dataset is shown in Table 4.

**Table 4.** Discrimination and optimism of external validated models.

Models and model prediction		True label (frailty/nonfrailty)	AUC^a^, mean (95% CI)		Predictive values		Sensitivity	Specificity
					Positive	Negative		
**Logistic regression**								
	Frailty	140/65	0.75 (0.71‐0.78)		0.68	0.80	0.73	0.76
	Nonfrailty	52/207				
**K-nearest neighbors**								
	Frailty	41/35	0.54 (0.51‐0.57)		0.54	0.61	0.21	0.87
	Nonfrailty	151/237						
**Random forest**								
	Frailty	137/63	0.75 (0.71‐0.78)		0.69	0.79	0.71	0.77
	Nonfrailty	55/209						
**Multilayer perceptron artificial neural network**								
	Frailty	98/38	0.68 (0.65‐0.72)		0.72	0.71	0.51	0.86
	Nonfrailty	94/234						
**Gradient boosting classifier**								
	Frailty	89/31	0.69 (0.65‐0.72)		0.74	0.70	0.46	0.89
	Nonfrailty	103/241						
**Linear support vector machine classifier**								
	Frailty	131/60	0.73 (0.70‐0.77)		0.69	0.78	0.68	0.78
	Nonfrailty	61/212				

a AUC: area under the receiver operating characteristic curve.

The overall discrimination performance was presented by mean AUC and 95% CI via 1000-fold bootstrapping. The LR and RF model achieved the highest overall performance with mean AUC 0.75 (95% CI 0.71‐0.78), followed by SVM (mean AUC 0.73, 95% CI 0.70‐0.77), and GBC (mean AUC 0.69, 95% CI 0.65‐0.72), respectively. The LR model had the highest sensitivity (73%) and the MLP model had the highest specificity (86%). KNN had the lowest discrimination performance with all metrics. Other metrics that were not affected by data rebalancing, like PPV and NPV, were also used to express the performance of the models. The best performance for PPV was found in the GBC model (0.74); for NPV, it was found in the LR model (0.80).

### Model Calibration

**Figure 2.**
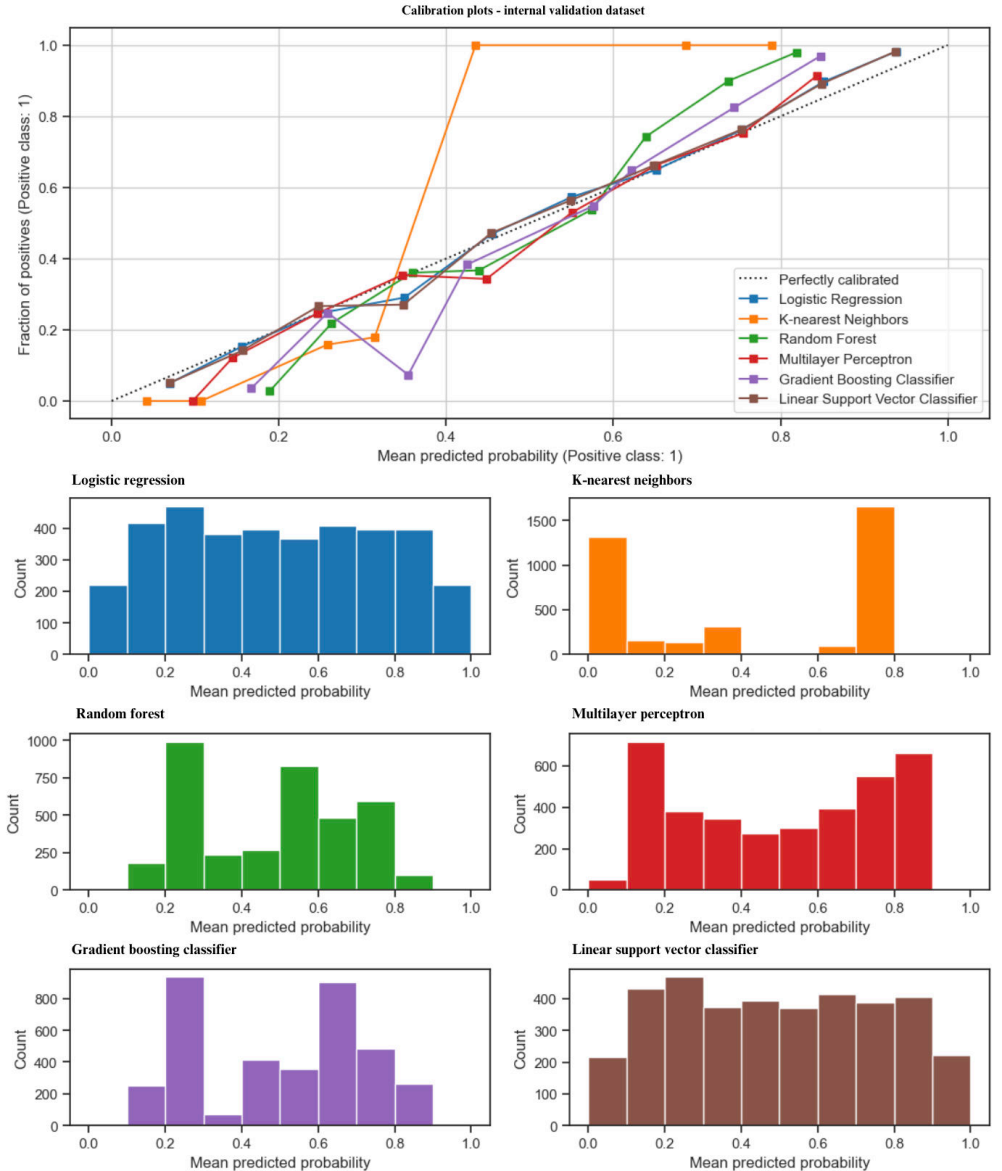
Model calibration of the developed models with internal validation.

The model calibration was visualized with the calibration plot, which compared the expected probability of frailty, and the mean 10-fold cross-validation predicted the probability of each model. In the internal validation dataset (Figure 2), the LR, SVM, and MLP were well-calibrated with the expected probability of the data. The distribution of predicted probabilities of the models was well-balanced between 0.00 and 1.00. The KNN classifier aligned well with the MPP between 0.00 and 0.40 but it overestimated the high predicted probability. In contrast, the GBC underestimated MPP between 0.20 and 0.40 but aligned well with the rest of the expected probability. These results suggest that LR, SVM, and MLP are the most reliable for balanced predictions, while KNN, GBC, and RF require careful consideration depending on the probability range of interest.

**Figure 3.**
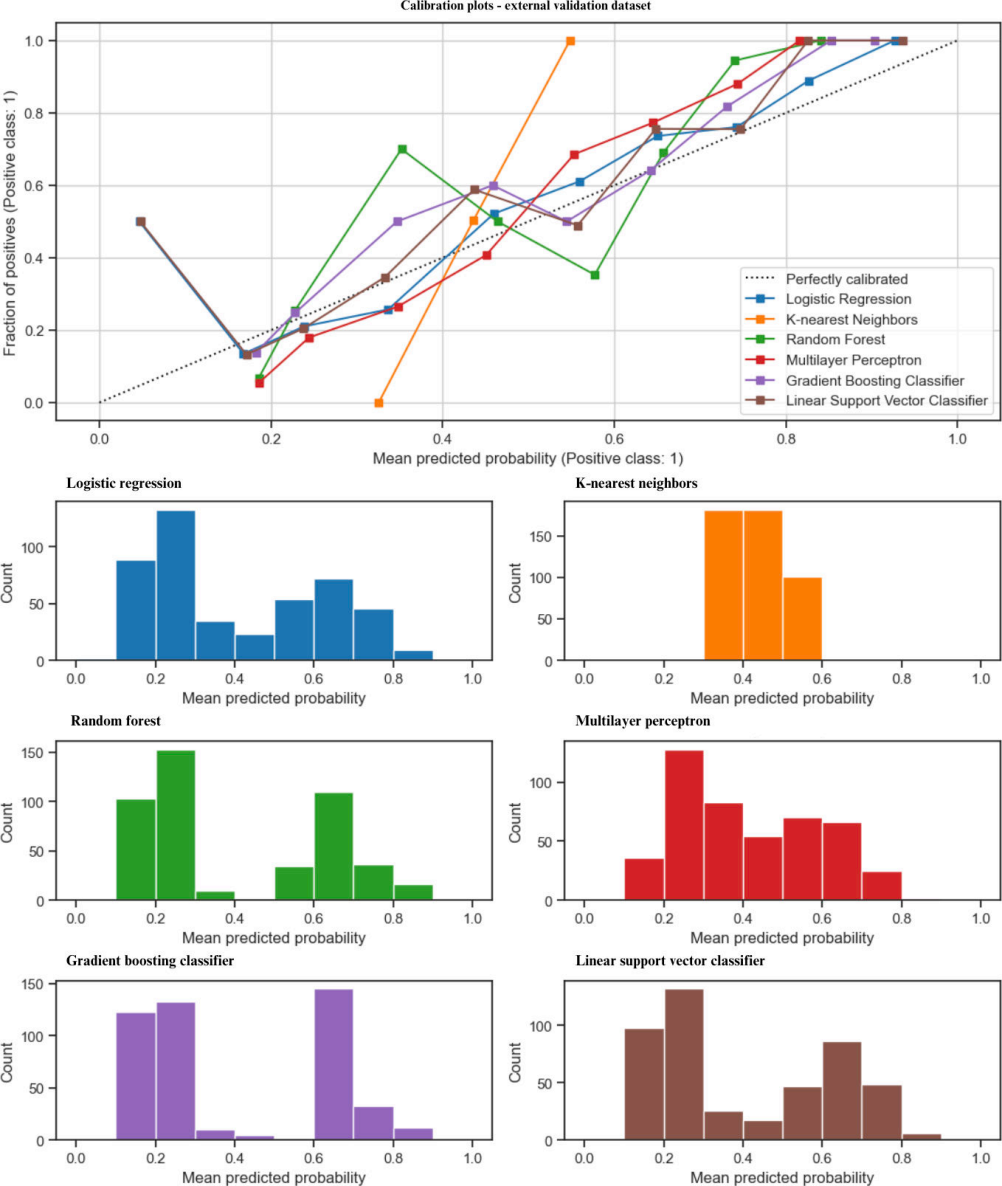
Model calibration of the developed models with external validation.

In the external validation dataset (Figure 3), the LR, SVM, and MLP were almost perfectly calibrated to the expected probability of the data with a slight overestimation in the 0.40‐0.80 MPP for MLP and an overestimation in the 0.00‐0.40 MPP for LR and SVM. These 3 models have a similarly balanced distribution of MPP. RF aligned poorly with an overestimation in the low and high MPP, and it underestimated in the range between 0.40 and 0.70. The GBC aligned well with the calibration plot after 0.60 but overestimated before 0.40. The KNN classifier was poorly calibrated to the plot, giving only MPP in the range of 0.30‐0.60.

## Discussion

### Model Performance

The internal validated models all showed remarkable discrimination performance, with the KNN model having the highest overall discrimination performance. However, the KNN model performed poorly in external validation datasets, indicating overfitting and the inability to generalize its results to other populations. In terms of model optimism, the LR, SVM, and MLP models had better discrimination capabilities, despite a slight decline in the models’ performance during external validation. The mean AUCs of these models remained in the range of fair to good performance. When considering all aspects of model performance, the LR model was the most preferable because it provided good discrimination (AUC), an optimal range of predicted probabilities, and good calibration in both the internal and external validation datasets. On the other hand, other models had predicted probabilities that were not extreme enough, resulting in poor to fair calibrations, and they lacked consistency between the internal and external validation datasets. In comparing the performance and validation aspects of previous frailty prediction models, most studies showed better performance than our best model (LR with a mean AUC of 0.81 in the internal validation set) [45]. The ELSA cohort study (2023) [46] reported the best performance from an RF model with an internal AUC of 0.92. Similarly, Bu et al (2023) [47] presented a multivariate LR model for predicting frailty in diabetic patients that had an AUC of 0.88 in the internal validation set. Additionally, a study on predictive modeling for frailty in older people using various machine learning methods found that artificial neural networks and SVM had the best performance in predicting mortality, with accuracies around 0.78‐0.79 [32]. Although these studies reported high internal performance metrics, the absence of external validation raises concerns about their generalizability, especially for our focus on the community-dwelling older adult population in general. Our LR showed a mean AUC of 0.75 for the external validation dataset, which dropped slightly from the internal validated results, demonstrating a better edge in the community setting for our focused population. Furthermore, the predictors included in the previously reported model played an important role in prediction performance. Particularly, the utilization of handgrip strength in the study conducted by Bu et al [47] and the assessment of balance and chair stand in the ELSA cohort study [46] could potentially explain the high reported performance in both studies. These predictors either formed part of the frailty phenotype or served as surrogates for physical performance. However, incorporating these predictors into the models necessitated a trade-off between their value added to model performance and the difficulties of model utilization due to the time and skill required for the assessments. In our study, we proposed more parsimonious models using simpler predictions, which still achieved satisfactory performance for frailty screening.

### Feature Selection and Findings Explanation

This study’s findings suggest that machine learning models can be effective in classifying frailty status among community-dwelling older adults in Northern Thailand using simple predictors including, age, gender, household living arrangement, underlying diseases (hypertension and dyslipidemia), BMI, waist and calf circumference, and level of exhaustion. Age, BMI, waist circumference, and calf circumference are all potent risk factors for frailty in older adults. When the human body deviates from the normal physiologic process of aging, our levels of estrogen and testosterone gradually decline. These hormones play a vital role in maintaining muscle and bone mass, enhancing strength, and promoting optimal nervous system function [48,49]. As a result, the aging process can accelerate the decline of muscle, bone, and the nervous system, transforming an individual from fit to frail. Additionally, the female population is at higher risk of frailty because the normal bone turnover cycle is disrupted by estrogen deficiency during menopause, increasing bone resorption over deposition, and resulting in net bone loss in women [50]. Household living arrangement was also added to model features as we found that there were studies that showed an association of social adversity and support with frailty status [51,52]. Lastly, we selected level of exhaustion as a predictor in our models, as it had the highest crude aOR among all other features, and we found that it is highly feasible and time-efficient to acquire this data in real clinical settings, compared to other Fried’s phenotypes, which involve multiple anthropometric and physical performance tests. These predictors also showed a significant contribution to the frailty prediction in the previous studies [46,47]. Most of our features used in the model are easily collectible, which makes it highly feasible and time-efficient to acquire all data in real clinical settings without depending on a high-level professional, making our model highly applicable for early frailty screening.

### Limitations

The most important limitation of this study’s models is the generalizability of the models. Our internal and external validation datasets were collected from a community-dwelling population in Lampang and Chiang Mai, respectively, which we confidently believe means that our models have high generalizability to the general population in Thailand. From our perspective, we assume that the models could also be implemented in other Asian countries, as studies showed similar frailty prevalence and population characteristics such as anthropometric measurements, age, household living arrangement, and underlying diseases of hypertension and diabetes [53-58].

However, we encourage conducting validation studies for other regions and populations before clinical application as frailty risk factors do vary across countries as well as in regions with different socioeconomic and health care contexts [59-61]. Another limitation of our study is spectrum bias, as our models were only able to distinguish between frail and robust older adults, despite Fried’s criteria having 3 stages of frailty: robust, prefrail, and frail [62]. Nonetheless, we do not believe this bias will significantly affect our study’s primary objective, which is the early detection of frailty in older adults to effectively prescribe nutrition and exercise interventions.

### Implications

Identifying frailty early helps patient gain access to interventions like nutritional support and exercise programs faster, improving outcomes for older adults by preventing frailty progression, reducing falls, hospitalizations, and mortality, and enhancing quality of life [63,64]. We see 2 possibilities for the practical application of our developed and validated models. One option is to develop a web application that serves as a frailty screening tool that could be self-assessed by individuals or be used in outpatient clinic settings to screen patients. This would help health care providers efficiently identify patients with frailty who require closer monitoring or interventions. We have implemented the validated models to run on our web application, which can be accessed at the link in the Data Availability section.

Another option is to incorporate our machine learning models into electronic medical record or health surveillance systems. The machine learning models could be integrated with the electronic medical record system to provide automated frailty probability scores for each patient. This would enable health care professionals to identify patients who require closer monitoring or interventions and could help optimize treatment plans for the community-dwelling population at risk of frailty. Furthermore, this could help future researchers retrieve and analyze frailty data from the hospital easier, leading to a better understanding of the factors that contribute to frailty and the development of more effective interventions, which also promotes more efficient use of resources within the health care system.

### Conclusion

Machine learning models were fairly good at classifying frailty status among Thai community-dwelling older adults using age, gender, household living arrangement, underlying diseases (hypertension and dyslipidemia), BMI, waist and calf circumference, and level of exhaustion as predictors. The LR and RF models demonstrated the best discrimination performance and model calibration in both the internal and external validation datasets.

There are 2 potential practical applications for utilizing the study findings. These include creating a web application for self-screening or individual screening and incorporating machine learning models into electronic medical record or surveillance systems to provide automated frailty probability scores for individual patients. We advocate for further research on model external validation and temporal recalibration to enhance the model’s practicality and applicability to the specific context in which it is used.

## Supplementary material

10.2196/62942 Multimedia Appendix 1 Supplementary materials

## Abbreviations

aOR: adjusted odds ratio
AUC: area under the receiver operating characteristic curve
CC: calf circumference
GBC: gradient boosting classifier
KNN: k-nearest neighbors
LR: logistic regression
MLP: multilayer perceptron artificial neural network
MPP: mean predicted probability
NPV: negative predictive value
PPV: positive predictive value
RF: random forest
SMOTE: synthetic minority oversampling technique
SVM: linear support vector machine classifier
TRIPOD+AI: Transparent Reporting of a multivariable prediction model for Individual Prognosis Or Diagnosis plus Artificial Intelligence
