# Correction: Machine Learning Models for Frailty Classification of Older Adults in Northern Thailand: Model Development and Validation Study

**DOI:** 10.2196/75690

**Published:** 2025-04-22

**Authors:** Natthanaphop Isaradech, Wachiranun Sirikul, Nida Buawangpong, Penprapa Siviroj, Amornphat Kitro

**Affiliations:** 1 Department of Community Medicine, Faculty of Medicine, Chiang Mai University, 110, Intrawarorot Road, Meaung, 50200, Thailand, 66 53935472, 66 935476; 2 Environmental and Occupational Medicine Excellence Center, Faculty of Medicine, Chiang Mai University, Chiang Mai, Thailand; 3 Center of Data Analytics and Knowledge Synthesis for Health Care, Faculty of Medicine, Chiang Mai University, Chiang Mai, Thailand; 4 Department of Biomedical Informatics and Clinical Epidemiology, Faculty of Medicine, Chiang Mai University, Chiang Mai, Thailand; 5 Department of Family Medicine, Faculty of Medicine, Chiang Mai University, Chiang Mai, Thailand

**Keywords:** aged care, gerontology, geriatric, old, aging, clinical decision support, delivering health information and knowledge to the public, diagnostic systems, digital health, epidemiology, surveillance, diagnosis, frailty, machine learning, prediction, predictive, AI, artificial intelligence, Thailand, community dwelling, health care intervention, patient care

In “Machine Learning Models for Frailty Classification of Older Adults in Northern Thailand: Model Development and Validation Study” (JMIR Aging 2025;8:e62942) one error was noted.

Reference 44 was a duplicate of reference 36, which reads as follows:

*Thinuan P, Siviroj P, Lerttrakarnnon P, Lorga T. Prevalence and potential predictors of frailty among community-dwelling older persons in northern Thailand: a cross-sectional study. Int J Environ Res Public Health. Jun 8, 2020;17(11):4077.*

Reference 44 has therefore been removed, and all subsequent references have been reordered accordingly.

The correction will appear in the online version of the paper on the JMIR Publications website, together with the publication of this correction notice. Because this was made after submission to PubMed, PubMed Central, and other full-text repositories, the corrected article has also been resubmitted to those repositories.

