# Supplementary material for: Machine Learning Models for Frailty Classification of Older Adults in Northern Thailand: Model Development and Validation Study
Source: JMIR Aging. 2025 Apr 2;8:e62942. doi: 10.2196/62942 (PMC12038762; doi:10.2196/62942)
Supplement: Multimedia Appendix 1 [file aging-v8-e62942-s001.docx]

**Table S1.** The parameter setting of the derived models

| **Models** | **Parameters Settings** |
| --- | --- |
|  |  |
| **LR** | {C=3.1, class_weight="balanced", penalty="l2", solver="liblinear"} |
| **KNN** | {algorithm="auto", metric="manhattan", n_neighbors=2, weights="uniform"} |
| **RF** | {bootstrap=False, class_weight="balanced_subsample", criterion="gini",max_depth=3, max_features="sqrt", n_estimators=50} |
| **MLP** | { activation="tanh", hidden_layer_sizes=[60], learning_rate="invscaling", solver="adam")} |
| **GBC** | {criterion="friedman_mse", learning_rate=0.1, loss="exponential", max_depth=1,max_features="sqrt", min_weight_fraction_leaf=0.0, n_estimators=20, subsample=1.0} |
| **SVM** | { C=1, dual=False, loss="squared_hinge", multi_class="ovr", penalty="l2"} |

Logistic Regression (LR), K-nearest Neighbors (KNN), Random Forest (RF), Multilayer Perceptron (MLP), Gradient Boosting Classifier (GBC), Linear Support Vector Classifier (SVM)
